# Effects of repeated waist-pull perturbations on gait stability in subjects with cerebellar ataxia

**DOI:** 10.1186/s12984-019-0522-z

**Published:** 2019-04-11

**Authors:** Federica Aprigliano, Dario Martelli, Jiyeon Kang, Sheng-Han Kuo, Un J. Kang, Vito Monaco, Silvestro Micera, Sunil K. Agrawal

**Affiliations:** 1 The BioRobotics Institute, Scuola Superiore Sant’Anna, Pisa, Italy; 2 Department of Mechanical Engineering, Columbia University, New York, NY USA; 3 Department of Neurology, College of Physicians and Surgeons, Columbia University Medical Center, New York, NY USA; 4 Division of Movement Disorders, Department of Neurology, Columbia University Medical Center, New York, NY USA; 5 Bertarelli Foundation Chair in Translational Neuroengineering, Center for Neuroprosthetics and Institute of Bioengineering, School of Engineering, École Polytechnique Federale de Lausanne (EPFL), Lausanne, Switzerland

**Keywords:** Gait perturbations, Cerebellar ataxia, Balance recovery, Perturbation-based training

## Abstract

**Background:**

Damage to the cerebellum can affect neural structures involved in locomotion, causing gait and balance disorders. However, the integrity of cerebellum does not seem to be critical in managing sudden and unexpected environmental changes such as disturbances during walking. The cerebellum also plays a functional role in motor learning. Only a few effective therapies exist for individuals with cerebellar ataxia. With these in mind, we aimed at investigating: (1) corrective response of participants with cerebellar ataxia (CA) to unexpected gait perturbations; and (2) the effectiveness of a perturbation-based training to improve their dynamic stability during balance recovery responses and steady walking. Specifically, we hypothesized that: (1) CA group can show a corrective behavior similar to that of a healthy control group; (2) the exposure to a perturbation-based treatment can exploit residual learning capability, thus improving their dynamic stability during balance recovery responses and steady locomotion.

**Methods:**

Ten participants with cerebellar ataxia and eight age-matched healthy adults were exposed to a single perturbation-based training session. The Active Tethered Pelvic Assist Device applied unexpected waist-pull perturbations while participants walked on a treadmill. Spatio-temporal parameters and dynamic stability were determined during corrective responses and steady locomotion, before and after the training. The ANalysis Of VAriance was the main statistical test used to assess the effects of group (healthy vs CA) and training (baseline vs post) on spatio-temporal parameters of the gait and margin of stability.

**Results:**

Data analysis revealed that individuals with cerebellar ataxia behaved differently from healthy volunteers: (1) they retained a wider base of support during corrective responses and steady gait both before and after the training; (2) due to the training, patients improved their anterior-posterior margin of stability during steady walking only.

**Conclusions:**

Our results revealed that participants with cerebellar ataxia could still rely on their learning capability to modify the gait towards a safer behavior. However, they could not take advantage from their residual learning capability while managing sudden and unexpected perturbations. Accordingly, the proposed training paradigm can be considered as a promising approach to improve balance control during steady walking in these individuals.

## Background

Damage to the cerebellum can affect the neural structures involved in locomotion causing balance disorders, abnormalities in the execution of movements, and gait ataxia [1–4]. The gait of subjects affected by cerebellar diseases is characterized by increased step width and variability, abnormal oscillations of the trunk, uncoordinated walking patterns, irregular foot trajectories, and reduced stability [5–8]. All these symptoms are directly correlated with falls [9–12] according to the evidence that 84% of patients affected by degenerative cerebellar ataxia fall at least once in a year with traumatic consequences [13].

Several studies have investigated the role of the cerebellum during corrective responses during unexpected perturbations [14–17]. It has been argued that the integrity of the cerebellum is not critical for managing sudden environmental changes such as altering the speed of a split-belt treadmill [16] or applying disturbances while standing [15, 17] as individuals with cerebellar ataxia can react to unexpected perturbations [17]. Accordingly, previous literature corroborates the hypothesis that corrective responses, mainly driven by fast reflexes, are predominantly controlled by lower neural centers such as the spinal cord or the brainstem [16–19] and are not significantly influenced by the cerebellum.

On the other hand, it is well known that the cerebellum plays a functional role in predictive motor adaptations and motor learning [3]. Indeed, for many years, cerebellar ataxia was not considered as a treatable disease since subjects with cerebellar damages cannot adjust the predictive component of their movements during both upper and lower limbs related tasks [16, 20–22]. In spite of this, recent investigations suggest that subjects with cerebellar damages have enough learning capacity to benefit from intensive rehabilitation treatments based on static and dynamic balance and coordination exercises, particularly individuals with degenerative ataxia [23, 24]. For instance, subjects with progressive ataxia due to cerebellar degeneration who completed a 4-week intensive coordinative training showed improved motor performance and reduced ataxia symptoms measured by both quantitative movement analysis and clinical scores [25]. Research in this field is increasing, albeit the intensity, duration and content of the rehabilitation programs, and the neural mechanisms and substrates underlying the training effects in ataxias remain open to debate [23, 26, 27].

To summarize, the literature suggests that: (1) corrective motor behaviors are not expected to be significantly affected by cerebellar damages, since these are mainly mediated by moto-neuronal circuits belonging to the lower part of the central nervous system [16, 17]; and (2) individuals with cerebellar ataxia still retain residual learning capabilities [23]. Accordingly, if these individuals underwent a training program to improve their motor strategy against falls, they may show improved balance control.

Thus, the first aim of this study was to investigate the corrective response of participants affected by cerebellar ataxia. The biomechanical behavior of these individuals was analyzed when unexpected force perturbations were delivered to them during steady walking. In particular, since cerebellar ataxia is not expected to affect corrective motor behaviors [7, 16], we hypothesized that these participants would adopt similar compensatory strategies for balance recovery, when compared to able-bodied individuals.

The second aim of this study was to investigate whether the proposed training will improve the dynamic stability of participants with cerebellar ataxia, assessed by the Margin of Stability, during balance recovery and gait. This approach can be used to reduce the risk of fall during daily activities.

Several studies have demonstrated that a training program with balance perturbations could affect the neuromuscular skills required during fall prevention [28, 29]. The underlying rationale is that exposure to repeated perturbations could lead to motor adaptations that could improve the overall response under new perturbations [30]. As a consequence, participants may show improved recovery after unexpected loss of balance encountered in daily life and consequently reduce their falls risk. Accordingly, our hypothesis was that participants with cerebellar ataxia could benefit from an intensive perturbation-based intervention since they retain learning capability [23, 25].

The approach proposed in this study has been adopted to investigate neuromuscular adaptations in healthy (young and older adults) and parkinsonian subjects [30–33]. Results revealed that unexpected waist-pulls in different directions induce acute changes in gait and balance. Further, participants improved their gait stability during normal walking after the training and adapted their response to new perturbations. These findings are encouraging as higher gait stability is associated with reduced risk of falls.

## Methods

### Participants

Eighteen volunteers enrolled to participate in a study that included eight non-impaired participants (4 females) representing the healthy control group (HG) and ten individuals (3 females) with a clinical diagnosis of cerebellar ataxia (CA). For both groups, participant characteristics are reported in Table 1. They were informed about the research procedures and they signed a written consent approved by the Institutional Review Board of Columbia University.

**Table 1:** Characteristics (mean ± standard deviation) of subjects with cerebellar ataxia (CA) and healthy group (HG). *P*-values lower than 0.05 are in bold

	CA	HG	*p*-values (t-test for independent samples)
Age [yr]	51.9 ± 6.5	55.8 ± 8.3	0.29
Weight [kg]	82.7 ± 17.5	68.9 ± 11.2	0.07
Height [m]	1.77 ± 0.12	1.71 ± 0.1	0.24
Walking speed [m/s]	0.87 ± 0.13	0.95 ± 0.10	0.16
SARA score	6.0 ± 1.7	–	–

Participants with cerebellar ataxia were evaluated by an ataxia specialist. Exclusion criteria were the use of walking aids, presence of visual or cognitive impairments, presence of frank dystonia, myoclonus, or sensory neuropathy that might influence walking. Before testing, they underwent a thorough neurological examination to ensure that the muscle power was 5/5 throughout and there was no sensory neuropathy. The diagnosis of cerebellar ataxia was made by the presence of typical oculomotor findings, hand and leg dysmetria, ataxic gait with a wide-base, and variable stride length and direction with a subsequent confirmation of cerebellar atrophy in neuroimaging studies. The Scale for the Assessment and Rating of Ataxia (SARA) was used to evaluate severity of cerebellar ataxia ([34, 35]; see Tables 1 and 2). SARA has eight items related to gait, stance, sitting, speech, finger-chase test, nose-finger test, fast alternating movements and heel-shin test. SARA score increases with ataxia disease stage. Based on this score, participants with cerebellar ataxia displayed relatively mild functional deficits (SARA score is 6.0 ± 1.7 across participants in the eight tasks), and their characteristics were not different from those reported for healthy controls (*p* > 0.05, Table 1). Moreover, for each participant with cerebellar ataxia further details are reported in Table 2.

**Table 2:** Characteristics for each participant with cerebellar ataxia

Subject	Age [yr]	Gender	Treadmill walking speed [m/s]	Diagnosis (age at onset [yr])	Scale for the Assessment and Rating of Ataxia
CA_01	51	M	0.9	Idiopathic (41)^a^	6.5
CA_02	50	M	1.0	SCA 1 (39)	6.5
CA_03	66	M	0.8	Idiopathic (51)^a^	5
CA_04	58	M	0.7	MSA-C (56)	8
CA_05	43	M	0.8	MSA-C (41)	5
CA_06	48	F	1.1	SCA 1 (43)	7.5
CA_07	48	F	0.8	MSA-C (46)	8.5
CA_08	49	F	0.9	FA (late teen)	12.5
CA_09	56	M	0.7	SCA 3 (50)	6.5
CA_10	50	M	0.95	MSA-C (49)	5.5

^a^Idiopathic cases here were tested negative for repeat expansions of common genetic ataxias: Friedrich’s ataxia, spinocerebellar ataxia type 1, 2, 3, 6, 7, 10, 12, 17, and dentatorubral-pallidoluysian atrophy. *SCA 1* spinocerebellar ataxia type 1, *SCA 3* spinocerebellar ataxia type 3, *MSA-C* multiple system atrophy, cerebellar type, *FA* Friedreich’s ataxia

### Experimental setup and protocol

**Fig. 1:**
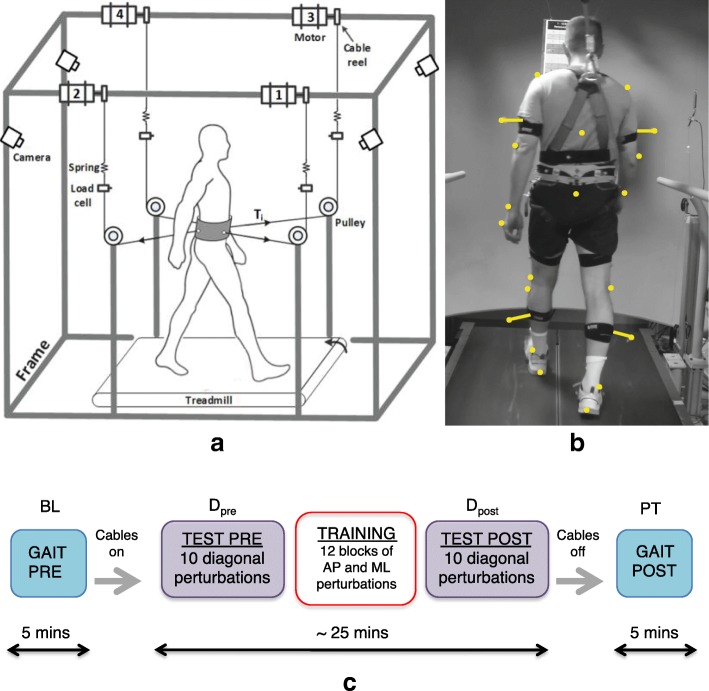
Experimental setup and protocol. **a** Schematic of the Active Tethered Pelvic Assist Device (A-TPAD). Antero-Posterior perturbations were applied using Motors 1 and 3 or Motors 2 and 4, Medio-Lateral perturbations were applied using Motors 1 and 2 or Motors 3 and 4. **b** Participant walks on the treadmill wearing the brace attached to the cables and the safety harness. Few reflective markers are highlighted in yellow dots. Full marker-set involves: C7 vertebrae, T10 vertebrae, clavicle, sternum and acromions, for the upper trunk; lateral and medial epicondyle of the humerus, radial and ulnar styloids, third metacarpal bones and additional markers rigidly attached to wands over the midhumerus, for both arms; anterior superior iliac spines and sacrum, for the pelvis; greater trochanters external surface, lateral and medial epicondyle of the femurs, heads of the fibula, lateral and medial malleolus, calcaneus, first and fifth metatarsal heads, toe, and additional markers rigidly attached to a wand over the midfemurs and midshaft of the tibia, for both legs; 4 markers attached to the brace. **c** Experiment protocol

Experimental sessions were carried out using a modified version of a cable actuated pelvic device, referred to as Active Tethered Pelvic Assist Device (A-TPAD; Fig. 1a). Details of its design and control can be found also in [31, 36].

Four AC servo motors (Kollmorgen, AKM21E) were mounted on an inertial rigid frame and were connected through cables to a hip belt worn by the participant. Cables were routed using pulleys and were diagonally directed away from the participant (Fig. 1a and b). Waist-pull perturbations were provided by applying a transient tension pulse in one or two of these four cables while the participant walked on a split-belt treadmill (Bertec, Instrumented Treadmill). This treadmill was instrumented with two three-dimensional force plates on each side of the split belt in order to measure the ground reaction forces and detect in real-time gait events such as heel strikes (threshold set at 50 N), enabling perturbations in a repeatable and controlled way. The tension in each cable was monitored by a load cell (Transducer techniques, MLP 200) that was installed in series between the pelvic belt and the electrical motor.

When cables were attached to the participants, a constant tension of 25 N was applied by each motor as the minimum tension to prevent cables from slacking. Perturbations were provided along the Antero-Posterior (AP) or Medio-Lateral (ML) directions by applying a transient pulse using two of the four cables. Specifically, suitable cable tension values were applied to each motor to apply a resultant force of desired magnitude and direction. To do this, before the experiment, a calibration trial was performed: 30-s walking was performed and cable attachment locations (on the subject and on the fixed reference frame) at each time point of interest (i.e., right and left heel strikes) were recorded and averaged between steps. The desired tension values for each motor to achieve a force in the AP or ML direction was computed based on the angles each cable formed with respect to the fixed frame.

Figure 1c shows the experimental protocol. Before each experiment, preferred walking speed was determined by gradually increasing the speed by 0.1 m/s until the participant reported that the speed was too fast and then reducing it by 0.1 m/s. Once determined, the walking speed was maintained constant during the experiment.

Initially, cables were not attached to the brace and participants walked on the treadmill at their preferred walking speed for 5 min. Data collected during this session was used as a reference in the analysis and was labeled as baseline.

Then, the four cables were attached to the brace. During the test sessions, before and after the training session (D_pre_ and D_post_, respectively), all participants were tested with 10 perturbations (5 repetitions along the two diagonal perturbations). Perturbations consisted of a pull of 15% of the participants’ body weight (BW) with Motor 2 (diagonally back on the right) at right heel strike or a pull of 15% BW with Motor 4 (diagonally back on the left) at left heel strike. The first perturbation was delivered at right heel strike and the order of perturbations was alternated.

During the training session, participants were trained with 12 blocks of eight different AP and ML perturbations delivered at heel strike, as reported in [31]. Specifically, at the beginning of the training, the peak force was 15% BW for AP and 5% BW for ML perturbations, respectively. After every four blocks, the peak perturbation magnitude was increased by 5% BW. The number of steps between perturbations was randomized (4–15 steps between perturbations) as well as the order of perturbations in each block. The duration of training and test sessions was approximately 25 min. Participants were aware that they could be perturbed at the waist when the cables were attached, but were not informed about the magnitude, the direction, or the timing of the perturbations. All perturbations were delivered while walking at a constant speed and consisted of a trapezoidal force profile (rise, hold and fall, each lasting 150 ms). Finally, cables were removed and participants walked for another 5 min. Data collected during this session were used in the analysis and were labeled as post-training.

In order to reduce the risk of fatigue, the treadmill was stopped every block during the training session and participants were told that they could rest for any length of time if they felt tired. For the duration of the experiment, participants wore an overhead safety harness to prevent them from falling while the harness did not restrict their movements (Fig. 1b). In addition, handrails were mounted on the platform to help participants feel at ease - participants were instructed to use these only if needed.

To monitor participants’ movement during the experimental trials, whole body kinematics were recorded at 200 Hz using a 10-camera motion capture system (Vicon Bonita-10 series). In particular, a full body marker-set was defined involving 49 reflective markers attached to the participants, as described in Fig. 1b.

### Data analysis

All data analysis was performed in Matlab (The MathWorks, Inc., Natick, MA, USA). Marker trajectories were low-pass filtered at a cut-off frequency of 10 Hz using a fourth-order zero-lag Buttherworth filter. Timings of main foot events, i.e., heel strike and toe off, were estimated from the local minimum vertical positions of the heel and toe markers, respectively. A 14-segment model (i.e., upper trunk, upper arms, forearms, hands, pelvis, thighs, shanks and feet) was developed to calculate the Center of Mass (CoM) of the whole body. Specifically, for each body segment, inertial parameters (i.e., mass and center of mass position) and joint center locations were chosen in accordance with [37]. The CoM was calculated from the weighted sum of the 14-segment model.

Spatio-temporal parameters were calculated during unperturbed walking trials (baseline and post-training) and balance recovery responses (test sessions, D_pre_ and D_post_), according to literature [18, 19]. Step length and width were calculated as the AP and ML displacements between consecutive right and left heel strikes, respectively. Step time was evaluated as the interval between two consecutive right and left heel strikes. Stance duration, described as a percentage of the gait cycle (stance_%_), was defined as the period during which the right foot was on the ground (i.e., from the heel strike to the consecutive toe off).

Dynamic stability was assessed during unperturbed walking trials (baseline and post-training) and balance recovery responses (test sessions, D_pre_ and D_post_) using the forward and lateral Margin of Stability (MoS) [38]. The MoS is given by the difference between the anterior boundary of the Base of Support (BoS) and the Extrapolated Center of Mass position (XCoM). The AP and ML components of the XCoM were computed as:

$$ {XCoM}_{AP, ML}={CoM}_{AP, ML}+\frac{{\dot{CoM}}_{AP, ML}}{\sqrt{\frac{g}{h_{CoM}}}} $$

where *CoM*_*AP*, *ML*_ and ${\dot{CoM}}_{AP, ML}$ are the AP and ML position and velocity of the *CoM*, *h*_*CoM*_ is the estimated pendulum length based on the height of the *CoM* during standing and *g* is the gravitational acceleration (9.81 m/s^2^). The AP and ML components of the MoS were calculated in a specific event (i.e., at the heel strike of the left foot). Accordingly, both components of the BoS were estimated at heel strike in a double support configuration using the toe markers from both feet.

During walking trials (baseline and post-training), spatio-temporal parameters were calculated during 10 gait cycles, and AP and ML components of MoS were calculated during 10 heel strikes. Then, for each subject and trial (baseline and post-training), the mean value of each variable was calculated as the average value among all counted steps.

During the test sessions (D_pre_ and D_post_), spatio-temporal parameters were calculated during the compensatory cycle (i.e., from the perturbation onset – right heel strike – to the subsequent left heel strike), and AP and ML components of MoS were assessed at the end of each compensatory step (i.e., at the first heel strike immediately after the perturbation onset). Only data relative to disturbances delivered at the right heel strikes were taken into account for data analysis.

### Statistical analysis

To investigate possible differences between two groups regarding their characteristics (i.e., age, body weight, body height and walking speed), a t-test for independent samples was used.

For each phase of the experimental protocol, all outcome variables (spatio-temporal parameters and AP and ML components of MoS) were used as dependent measures.

To test the effects of trial (2 levels) and group (2 levels: CA and HG) on both the acute responses to perturbations (levels: D_pre_ and D_post_), and walking (levels: baseline and post-training), the two-way repeated measures ANalysis Of VAriance (ANOVA) was performed. If significant, the main effect of these analyses was followed up by pairwise comparisons with Bonferroni’s correction. Comparisons were made between the two levels of trials (D_pre_ and D_post_ or baseline and post-training) and groups (CA and HG). Statistical significance was set at *p* ≤ 0.05.

## Results

### Balance recovery responses after perturbations

**Fig. 2:**
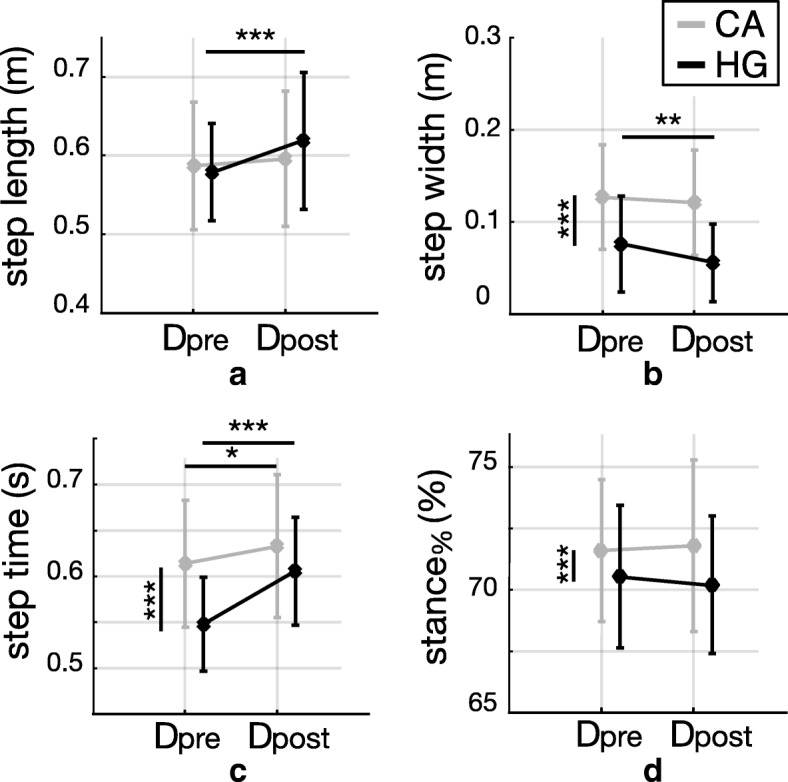
Spatio-temporal parameters during balance recovery responses. Spatio-temporal parameters (mean ± standard deviation) observed during balance recovery responses after diagonal perturbations, before (D_pre_) and after (D_post_) training, for subjects with cerebellar ataxia (CA; gray bars) and healthy control group (HG; black bars). Pairwise comparisons reaching significance are reported (* *p* < 0.05, ** *p* < 0.01, *** *p* < 0.0001); vertical and horizontal lines are related to the group (CA vs HG) and session (pre vs post) effects, respectively. **a** step length; **b** step width; **c** step duration; **d** stance_%_

Spatio-temporal parameters are depicted for both groups (CA and HG), before (D_pre_) and after (D_post_) the perturbation-based training in Fig. 2. During balance recovery responses, step width, step time and stance_%_ showed a significant main effect of group (all *p*-values < 0.0001), while step length was not different between groups (*p* = 0.321; Fig. 2a). The CA group walked with a larger step width (Fig. 2b), higher step time (Fig. 2c) and stance_%_ (Fig. 2d) than HG.

The exposure to repeated perturbations during the training session produced the following modifications: step length (*p* = 0.001), step width (*p* = 0.016) and step time (*p* < 0.0001) showed significant main effects of the session. The stance_%_ was not affected by the perturbation-based training (*p* = 0.510; Fig. 2d). In addition, step time (*p* < 0.001) and step length (*p* = 0.037) demonstrated a significant interaction between session and group. Pairwise comparisons revealed that at D_post_ compared to D_pre_: (i) only the HG significantly increased their step length (*p* = 0.002; Fig. 2a) and decreased step width (*p* = 0.019; Fig. 2b); (ii) both groups increased step time (HG: *p* < 0.0001, CA: *p* = 0.045; Fig. 2c).

**Fig. 3:**
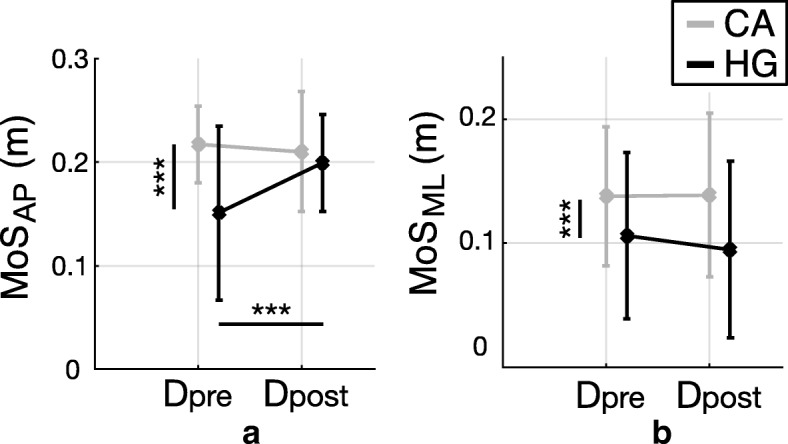
Margin of Stability during balance recovery responses. Dynamic stability (mean ± standard deviation) observed during balance recovery responses after diagonal perturbations, before (D_pre_) and after (D_post_) the training, for subjects with cerebellar ataxia (CA; gray bars) and healthy control group (HG; black bars). Pairwise comparisons reaching significance are reported (* *p* < 0.05, ** *p* < 0.01, *** *p* < 0.0001); vertical and horizontal lines are related to the group (CA vs HG) and session (pre vs post) effects, respectively. **a** MoS_AP_; **b** MoS_ML_

Dynamic stability, in terms of MoS_AP_ and MoS_ML_, is reported for both groups (CA and HG) before (D_pre_) and after (D_post_) the perturbation-based training session in Fig. 3.

Results showed that the AP component of MoS was significantly affected by the group factor (*p* < 0.0001), revealing that MoS_AP_ was higher for CA group than for HG (Fig. 3a). In addition, the MoS_AP_ showed a significant main effect of the session (*p* = 0.006) and interaction effect between session and group (*p* < 0.0001). Post-hoc analysis revealed that only HG increased the MoS_AP_ at D_post_ (*p* = 0.002). The ML component of MoS was significantly affected by the group factor (*p* < 0.0001) such that the MoS_ML_ was higher for individuals with CA than for HG (Fig. 3b). The perturbation-based training session did not affect the ML component of the MoS for both groups (*p* = 0.243; Fig. 3b).

### Unperturbed walking

**Fig. 4:**
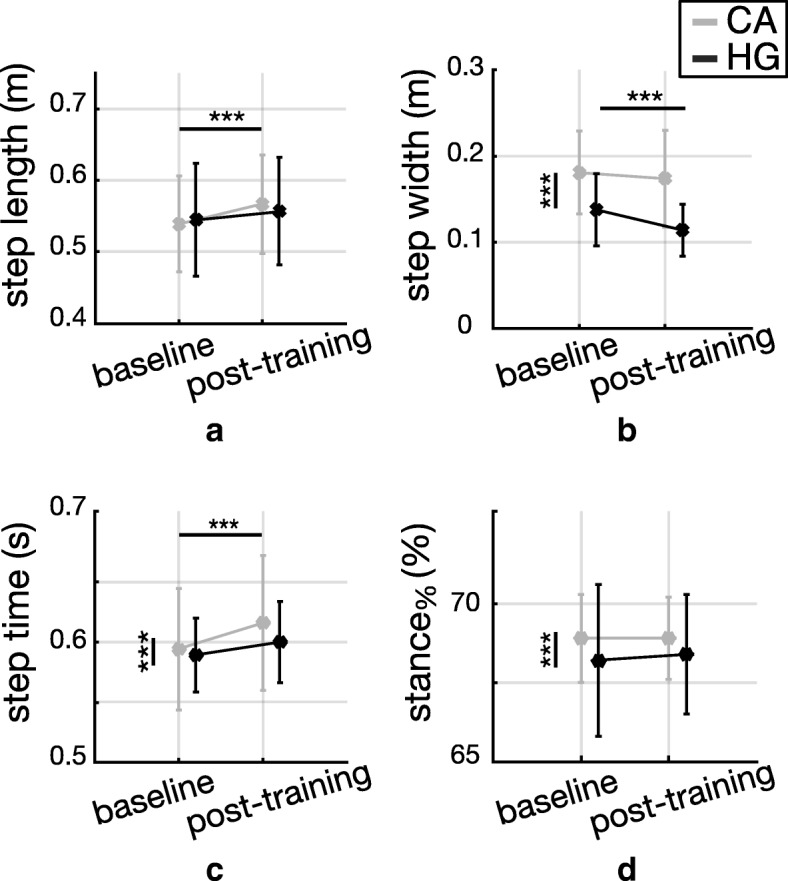
Spatio-temporal parameters during steady walking. Spatio-temporal parameters (mean ± standard deviation) observed during steady walking trials, before (baseline) and after (post-) training, for subjects with cerebellar ataxia (CA; gray bars) and healthy control group (HG; black bars). Pairwise comparisons reaching significance are reported (* *p* < 0.05, ** *p* < 0.01, *** *p* < 0.0001); vertical and horizontal lines are related to the group (CA vs HG) and session (pre vs post) effects, respectively. **a** step length; **b** step width; **c** step duration; **d** stance_%_

Figure 4 shows results about spatio-temporal parameters observed during unperturbed walking trials for both groups (CA and HG), before (baseline) and after the training session (post-training).

Step width (*p* < 0.0001), step time (*p* = 0.004), and stance_%_ (*p* < 0.0001) showed a significant main effect of group, such that participants with CA walked with a larger step width (Fig. 4b), and higher step time (Fig. 4c) and stance_%_ (Fig. 4d) than HG. Step length was not different between CA and HG groups during unperturbed walking trials (*p* = 0.752; Fig. 4a).

The perturbation-based training produced short-term after effects such that when cables were removed the gait pattern was modified. Indeed, step length, step width, and step time showed significant main effects of the session (all *p*-values< 0.0001). In addition, step length (*p* = 0.006) and step width (*p* = 0.008) had a significant interaction between session and group. Post-hoc analysis revealed that at post-training compared to baseline: (i) only HG decreased step width (*p* < 0.0001; Fig. 4b); (ii) only CA group increased step length (*p* < 0.0001; Fig. 4a) and step time (*p* = 0.0004; Fig. 4c).

**Fig. 5:**
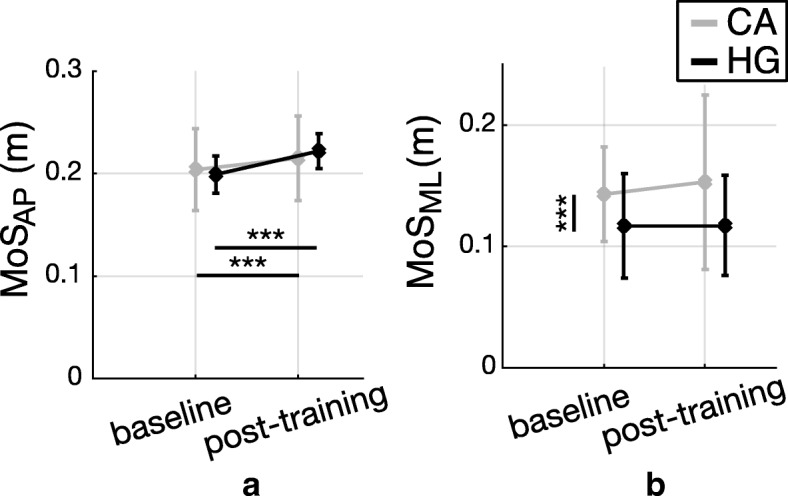
Margin of Stability during steady walking. Dynamic stability (mean ± standard deviation) observed during steady walking trials, before (baseline) and after (post-) training, for subjects with cerebellar ataxia (CA; gray bars) and healthy control group (HG; black bars). Pairwise comparisons reaching significance are reported (* *p* < 0.05, ** *p* < 0.01, *** *p* < 0.001); vertical and horizontal lines are related to the group (CA vs HG) and session (pre vs post) effects, respectively. **a** MoS_AP_; **b** MoS_ML_

Figure 5 shows results about the AP and ML components of MoS observed during unperturbed walking trials for both groups (CA and HG), before (baseline) and after the training session (post-training).

The AP component of MoS showed a significant main effect of the session (*p* < 0.0001) and significant interaction between session and group (*p* = 0.025). No differences were observed between groups (*p* = 0.616). Pairwise comparisons highlighted that both groups significantly increased their MoS_AP_ at post-training compared to baseline (Fig. 5a).

The ML component of MoS showed a significant main effect of group revealing that MoS_ML_ for individuals with CA was higher than that obtained for HG (*p* = 0.001; Fig. 5b). Differently, the MoS_ML_ did not show differences across the training session (*p* = 0.640; Fig. 5b), that is, the training did not modify the ML component of MoS, for both groups.

## Discussion

In this study, we investigated: (1) to what extent cerebellar ataxia affects the ability to manage unexpected perturbations during walking; (2) whether a perturbation-based training paradigm can produce short term aftereffects in CA group in improved balance control.

Our first hypothesis was that participants with cerebellar ataxia could adopt suitable compensatory strategies to counteract diagonal perturbations. Specifically, we expected that the counteracting behavior of CA group was comparable to those of HG, thus highlighting that the cerebellum does not play any significant functional role in balance recovery responses following unexpected disturbances. Results concerning balance control while managing diagonal perturbations revealed that participants with CA were characterized by different corrective responses compared to HG, in terms of both spatio-temporal parameters and margin of stability (Figs. 2 and 3). Based on current results, we reject our former hypothesis suggesting that the disease modifies the corrective behavior of participants.

Our second hypothesis was that participants with CA could benefit from the proposed training, thus showing improved balance control in both corrective response and unperturbed walking after the treatment. Results highlighted that the perturbation-based training led CA group to adapt their gait patterns toward a safer behavior, during unperturbed locomotion only (Fig. 5a); thus our latter hypothesis could not be rejected.

### Role of the cerebellum during balance recovery responses

Results highlighted that CA group was characterized by a wider step compared to healthy controls while managing unexpected diagonal perturbations and steady walking (Fig. 2b and Fig. 4b, respectively), both before and after the perturbation-based training, thus corroborating the outcome of previous studies [4, 23, 39–41]. In addition, the MoS in the frontal plane was larger in individuals with cerebellar ataxia (Fig. 3b and Fig. 5b), in accordance with literature [8]. Similarly, CA group had higher MoS_AP_ and step time than controls (Fig. 3a and Fig. 2c, respectively) during balance recovery responses elicited after diagonal perturbations, as already observed by other authors [42, 43]. According to these results, the training did not modify these behavioral features of CA group while managing unexpected perturbations (i.e., no significant effect of the session was observed for individuals with CA in Fig. 2b and Fig. 3b, as discussed in Section 4.2).

The greater MoS (both components) shown by subjects with CA while managing perturbations, mostly results from a greater base of support. It is important to highlight that CA group showed this biomechanical behavior since the beginning of the experimental session, i.e., outside the perturbation-based training (Fig. 3a and b). The greater MoS observed in participants with CA would not imply that their behavior is better (more stable) than that of HG. It would instead reflect a typical compensatory motor adaptation to lead impaired subjects to successfully perform the task and to prevent falling, as also observed in healthy people and individuals with neuro-musculoskeletal diseases [8, 43, 44]. Accordingly, the inherent larger base of support of CA group likely represents a neuromuscular adaptation following the disease and is adopted by these subjects to keep a safer balance.

The pathology-related neuromuscular adaptations shown by CA group, apart from the experimental conditions (i.e., walking vs managing perturbation, pre- vs post-training), did not allow us to unequivocally understand the contribution of the cerebellum during corrective behavior. In particular, we would have expected that the corrective response of CA group was comparable to that of healthy subjects, thus supporting the hypothesis that reactive motor behavior is predominantly controlled by lower neural centers (i.e., the cerebellar structures are not involved in reactive behaviors; see also [16, 17]). Our results instead showed that individuals with CA behaved differently than healthy participants. Accordingly, we are not allowed to reject the hypothesis that the cerebellum plays a functional role in balance recovery responses following unexpected perturbations. In this respect, further analyses are required to clarify the role of the cerebellum during reactive motor behaviors.

### Short term after-effects due to the proposed training paradigm

Concerning the short term adaptation during steady walking, our results revealed that participants with cerebellar ataxia, as much as healthy controls, significantly increased their balance control along the sagittal plane after the perturbation-based training (Fig. 5a). Specifically, the exposure to repeated waist-pull perturbations produced acute short term aftereffects in subjects with CA involving greater step length and duration, and greater MoS_AP_ (Fig. 4a and c and Fig. 5a). Regarding the frontal plane, both groups did not modify significantly their balance control along the ML direction (Fig. 5b), in accordance with previous findings [30].

These results, mainly relating to the sagittal plane, provide further support to the hypothesis that individuals affected by cerebellar ataxia still retain residual learning capabilities which can be enabled by choosing suitable training programs [18, 19]. In this respect and according to our results, a perturbation-based training program is expected to be a promising approach to allow these subjects to improve balance control during locomotion related motor tasks. Noticeably, a similar outcome was also observed in Parkinson’s patients [30]. Accordingly, we could speculate that the proposed training improves dynamic stability in steady walking in individuals affected by different kinds of neural diseases. However, this hypothesis deserves further research.

It is worth to observe that the perturbation-based training did not modify corrective balance recovery responses following diagonal perturbations in participants with cerebellar ataxia, while it did affect the corrective behavior of healthy control group (Figs. 2 and 3).

The absence of short term effects on corrective responses after a perturbation in CA group can be explained using the following two arguments. On one hand, the corrective motor response of individuals with CA was already altered due to the pathology-related neuromuscular adaptation, involving a wider step. Therefore, in spite of the perturbation-based paradigm, CA group could not further modify their counteractive strategy. Hence, the proposed training paradigm was not effective in reshaping their corrective motor behavior during reactive responses. On the other hand, unexpected perturbations mainly elicit motor behaviors encrypted in the lower part of the central nervous system [18, 19]. In this respect, our results suggest that cerebellar structures are not significantly involved while counteracting unexpected lack of balance, even if the pathology, per se, involves neuromuscular adaptation.

Summarizing, our results revealed that participants with cerebellar ataxia could still rely on their learning capabilities in order to modify volitional motor tasks (i.e., walking) toward a safer behavior. However, they could not take advantage from their learning capabilities while managing sudden and unexpected perturbations. Accordingly, we observed that even if a perturbation-based training can modify the stability of these subjects during walking-related motor tasks, it might not be effective to improve their counteracting behaviors following unexpected lack of balance.

It is intriguing to postulate the underlying mechanism of adaptation. On one hand, observed impaired subjects were affected by mild cerebellar atrophy. Accordingly, the adopted perturbation paradigm could have directly activated residual cerebellar capabilities thus improving patients’ behavior while walking unassisted. One the other hand, it is well known that multiple brain regions have roles in modulating the gait in addition to the cerebellum, including frontal lobes, parietal lobes, brainstem nuclei such as pedunculo pontine nuclei, and basal ganglia. Recent literature has indeed documented in depth the notion of compensatory effects of one brain region on another in gait abnormality of different neurodegenerative and neurodevelopmental disorders [45, 46]. Accordingly, we can speculate that the proposed perturbation paradigm could promote different mechanisms of brain plasticity in order to compensate for the impaired functions of the cerebellum. However, these possibilities deserve further explorations.

### Limits of the study

This study has some limitations. First, we do not know how long the gait improvement would last as we have not done extensive follow up in this preliminary study (only very acute effects were investigated – unperturbed walking was analyzed only for five minutes after the training). Second, the degree of improvement in cerebellar ataxia was measured by gait parameters and the translation of these into clinical improvements, such as reduction in the frequency of falls, needs to be further explored. Third, most of the participants have degenerative forms of cerebellar disorders, such as spinocerebellar ataxia and multiple system atrophy have additional brainstem involvement [47]. Brainstem has dense neuronal connections with the cerebellum; therefore, the roles of the brainstem versus the cerebellum in these motor paradigms cannot be easily separated. Future studies should focus on subjects with more discrete lesions on either the brainstem or the cerebellum to answer the region-specific effects within the cerebellar network. Fourth, the single training session could limit the generalization of these results. However, the strength of our study consists of investigating the effectiveness of a novel perturbation-based approach to improve cerebellar ataxia from the perspective of rehabilitation of gait and balance. Coordinated exercise has been shown to improve ataxia symptoms [24]. Our approach provides a more structured training regimen for those who have a higher risk of falls. In addition, quantitative measurements allowed us to investigate different aspects of gait and to probe the central nervous system for adaptation to random perturbations in future studies. Noticeably, this system could also provide training to subjects with cerebellar ataxia in “real life” situations in order to improve their general gait functions overall.

## Conclusions

This study suggests that the proposed perturbation-based training can be considered as a promising approach to improve balance control in subjects with cerebellar ataxia mostly with respect to unperturbed locomotion. In this respect, multiple sessions of such random perturbations could be a strategy for the gait rehabilitation of individuals with cerebellar ataxia.

## Abbreviations

ANOVA: ANalysis Of VAriance
AP: Antero-Posterior
A-TPAD: Active Tethered Pelvic Assist Device
BoS: Base of Support
BW: Body weight
CA: Cerebellar Ataxia
CoM: Center of Mass
D_post_: Diagonal perturbation after the training
D_pre_: Diagonal perturbation before the training
HG: Healthy Group
ML: Medio-Lateral
MoS: Margin of Stability
SARA: Scale for the Assessment and Rating of Ataxia
XCoM: Extrapolated Center of Mass
